# Changes in estimated glomerular filtration rate over time in South African HIV-1-infected patients receiving tenofovir: a retrospective cohort study

**DOI:** 10.7448/IAS.20.01/21317

**Published:** 2017-04-10

**Authors:** Reneé De Waal, Karen Cohen, Matthew P Fox, Kathryn Stinson, Gary Maartens, Andrew Boulle, Ehimario U Igumbor, Mary-Ann Davies

**Affiliations:** ^a^ Centre for Infectious Disease Epidemiology and Research, School of Public Health and Family Medicine, University of Cape Town, Cape Town, South Africa; ^b^ Division of Clinical Pharmacology, Department of Medicine, University of Cape Town, Cape Town, South Africa; ^c^ Departments of Global Health and Epidemiology, Boston University Center for Global Health and Development, Boston, United States of America; ^d^ Health Economics and Epidemiology Research Office, Department of Internal Medicine, School of Clinical Medicine, University of the Witwatersrand, Johannesburg, South Africa; ^e^ Médecins Sans Frontières, Khayelitsha, South Africa; ^f^ Division of Global HIV & TB, Center for Global Health, US Centers for Disease Control and Prevention, Pretoria, South Africa

**Keywords:** human immunodeficiency virus, tenofovir, kidney function, renal toxicity, antiretroviral pharmacovigilance

## Abstract

**Introduction**: Tenofovir has been associated with decline in kidney function, but in patients with low baseline kidney function, improvements over time have been reported. Additionally, the magnitude and trajectory of estimated glomerular filtration rate (eGFR) changes may differ according to how eGFR is calculated. We described changes in eGFR over time, and the incidence of, and risk factors for, kidney toxicity, in a South African cohort.

**Methods**: We included antiretroviral-naïve patients ≥16 years old who started tenofovir-containing antiretroviral therapy (ART) between 2002 and 2013. We calculated eGFR using the Modification of Diet in Renal Disease (MDRD), Chronic Kidney Disease Epidemiology Collaboration (CKD-EPI), and Cockcroft-Gault equations. We described changes in eGFR from ART initiation using linear mixed effects regression. We described the incidence of eGFR <30 mL/min on treatment, and identified associations with low eGFR using Cox regression.

**Results**: We included 15156 patients with median age of 35.4 years (IQR 29.9–42.0), median CD4 cell count of 168 cells/µL (IQR 83–243), and median eGFR (MDRD) of 98.6 mL/min (IQR 84.4–115.6). Median duration of follow up on tenofovir was 12.9 months (IQR 5.1–23.3).

Amongst those with a baseline and subsequent eGFR  available, mean eGFR change from baseline at 12 months was −4.4 mL/min (95% CI −4.9 to −4.0), −2.3 (−2.5 to −2.1), and 0.6 (0.04 to 1.2) in those with baseline eGFR ≥90 mL/min; and 11.9 mL/min (11.0 to 12.7), 14.6 (13.5 to 15.7), and 11.0 (10.3 to 11.7) in those with baseline eGFR <90 mL/min, according to the MDRD, CKD-EPI (n = 11 112), and Cockcroft-Gault (n = 9 283) equations, respectively.

Overall, 292 (1.9%) patients developed eGFR <30 mL/min. Significant associations with low eGFR included older age, baseline eGFR <60 mL/min, CD4 count <200 cells/µL, body weight <60 kg, and concomitant protease inhibitor use.

**Conclusions**: Patients on tenofovir with baseline eGFR ≥90 mL/min experienced small but significant declines in eGFR over time when eGFR was estimated using the MDRD or CKD-EPI equations. However, eGFR increased in patients with eGFR <90 mL/min, regardless of which estimating equation was used. Decreases to below 30 mL/min were uncommon. In settings with limited access to laboratory testing, monitoring guidelines should consider focusing on higher risk patients.

## Introduction

Tenofovir-containing antiretroviral therapy (ART) is recommended in local and international HIV treatment guidelines but is associated with kidney toxicity [1,2]. Several studies have shown modest, but significant, declines in kidney function over time in patients receiving tenofovir compared to those on other antiretrovirals [3–5]. A meta-analysis of five randomized controlled trials and six prospective cohort studies, with durations of follow up of 46–144 weeks, found that on average patients on tenofovir experienced a decline in estimated glomerular filtration rate (eGFR, calculated using the Cockcroft-Gault equation) of 3.92 mL/min more than patients not on tenofovir over the treatment period (n = 5 767, 95% confidence interval (CI) 2.13–5.70 mL/min) [3]. The studies included in the meta-analysis were mostly conducted in the United States or Europe, and the included patients had mean or median CD4 counts above 200 cells/mm^3^ in all but one study (range 197–516 cells/mm^3^), and mean or median baseline eGFR above 100 mL/min in all studies. In contrast, two studies conducted in Africa, in patients with median CD4 counts of 209 and 154 cells/mm^3^ respectively, showed that eGFR (Cockcroft-Gault) improved over time in patients on tenofovir, regardless of baseline kidney function [6,7]. A large Zambian cohort study, in patients with a median CD4 count of 151 cells/mm^3^, showed that while eGFR (calculated using the Chronic Kidney Disease Epidemiology Collaboration (CKD-EPI) equation) decreased by 15 mL/min by 12 months on treatment in those with normal kidney function at baseline (eGFR ≥90 mL/min), it improved by 30 mL/min in those with severely impaired baseline kidney function (eGFR <30 mL/min) [8].

Clinically significant tenofovir-related kidney toxicity is uncommon. The estimated incidence of decrease in creatinine clearance to below 50 mL/min (estimated using the Cockcroft-Gault equation) in patients on tenofovir, ranges from 3 to 8% in clinical trials and observational cohorts [6,7,9,10]. The estimated incidence of decrease in creatinine clearance to below 30 mL/min ranges from 0.2 to 1.6% [6,10,11], and of acute renal failure from 0.3 to 1.4% [12,13]. Risk factors for the development of renal impairment in patients on tenofovir include lower baseline CD4 count [5,14–16], lower baseline eGFR [14,17], older age [4,5,14–16], concomitant protease inhibitor use [4,5,9,18], lower body weight or body mass index [15,16], and comorbidities such as diabetes mellitus and hypertension [4,5,18].

Given the 2015 World Health Organization (WHO) recommendation to start ART regardless of CD4 count, and the fact that tenofovir has formed part of the WHO preferred first-line ART regimen since 2013, tenofovir use is likely to expand, including in many settings where laboratory monitoring is limited. WHO guidelines advise calculation of baseline eGFR if possible, especially in higher-risk patients, but state that laboratory monitoring on therapy is not compulsory [1]. The most efficient screening and monitoring strategy for resource-limited settings has not yet been determined [19]. Therefore, further studies are warranted to clarify the risk of tenofovir use in resource-limited settings.

Previous studies that have reported eGFR changes over time have used various estimating equations to calculate eGFR based on serum creatinine concentration. When compared to measured GFR, the CKD-EPI equation was found to be more accurate than the Modification of Diet in Renal Disease (MDRD) equation in HIV-infected patients on ART in studies conducted in the US and Europe [20–22], but it was not found to be superior in two South African studies in HIV-infected patients [23,24]. The magnitude and trajectory of eGFR changes over time in patients on tenofovir may differ according to which estimating equation is used: eGFR increased over time when estimated using the Cockcroft-Gault equation and decreased when using the MDRD and CKD-EPI equations in two studies conducted in the US and Puerto Rico, and in Senegal and Cameroon, respectively [25,26]. This suggests that differences between studies in observed eGFR changes over time might be due, at least in part, to the use of different estimating equations, as well as differences in the populations studied.

We describe changes in eGFR over time, and explore differences between three equations commonly used to estimate GFR, in HIV-1-infected patients receiving tenofovir-containing ART at two South African sites. We also describe the incidence of, and risk factors for, kidney dysfunction.

## Methods

We included antiretroviral-naïve patients who were at least 16 years old and who started tenofovir-containing ART between July 2002 and July 2013 at Khayelitsha, and between January 2005 and December 2012 at Themba Lethu. The Khayelitsha HIV Treatment Programme comprises three primary care clinics in Cape Town, South Africa, and was established in 2001 [27]. Themba Lethu clinic is based at a secondary level hospital in Johannesburg, South Africa, and was established in 2004 [28]. Each site has initiated over 20 000 adult patients on ART, and both sites follow standard ART and monitoring protocols published by the South African National Department of Health [2]. ART eligibility criteria changed during the period of this study, from a threshold CD4 count for ART initiation of <200 cells/µL before 2013 to <350 cells/µL from April 2013 [29,30]. Tenofovir has been part of the standard first-line ART regimen for patients 16 years and older without contraindications since 2010 [30]. The guidelines recommend screening for baseline kidney impairment before tenofovir initiation, and monitoring at 3, 6, and 12 months, then annually, by calculation of eGFR [2,30]. Both sites prospectively collect routine clinical data, such as demographic details, antiretroviral prescription, and laboratory results, electronically.

Creatinine concentrations were measured using an isotopic dilution mass spectrometry standardized assay. We calculated eGFR using the following equations: MDRD: eGFR (mL/min/1.73 m^2^) = 175 × (serum creatinine (Scr) (µmol/L)/88.4)^−1.154^ × (Age)^−0.203^ (× 0.742 if female) [31]; CKD-EPI: eGFR (mL/min/1.73 m^2^) = 144 × (0.993)^age^ × ((Scr (µmol/L)/88.4)/0.7)^−0.329^ (if female and Scr ≤62 µmol/L) or ((Scr (µmol/L)/88.4)/0.7)^−1.209^ (if female and Scr >62 µmol/L) or ((Scr (µmol/L)/88.4)/0.9)^−0.411^ (if male and Scr ≤80 µmol/L) or ((Scr (µmol/L)/88.4)/0.9)^−1.209^ (if male and Scr >80 µmol/L) [32]; and Cockcroft-Gault: eGFR (mL/min) = ((140-age) × weight × 1.228)/Scr (μmol/L) (× 0.85 if female) [33]. Although the MDRD and CKD-EPI equations include a factor for Black race, we did not adjust eGFR for race in this study for the following reasons: (i) the equations were derived in African American patients not Southern African patients [31,32] and South African studies have found that eGFR calculated without the race factor is more accurate when compared to measured GFR for both the MDRD [34,35] and CKD-EPI equations [23,24]; (ii) the study sites do not routinely collect race data. Weights were not always recorded on the same dates as creatinine concentrations, so we matched weights to creatinine concentrations to calculate Cockcroft-Gault eGFR. For baseline eGFRs, we matched the weight measured at the closest date to that of the creatinine concentration measurement if they were within 30 days of each other and both were measured between six months before and two weeks after ART initiation. For eGFRs up to 6 months on ART, we matched the weight measured at the closest date to that of the creatinine concentration measurement if they were within 14 days of each other. For eGFRs after 6 months on ART, we carried weights forward for up to 9 months to replace missing weights.

In those patients who had a baseline eGFR and at least one subsequent eGFR while on treatment available, we used linear mixed-effects regression to describe changes in eGFR from baseline (within 6 months before or 2 weeks after ART initiation) at 3, 6, 12, and 24 months after ART initiation. To assess whether mean changes from baseline were driven by individual patients stopping tenofovir if their eGFR decreased, we performed a sensitivity analysis restricted to those patients who remained on tenofovir for at least 1 year.

We summarized the proportion of patients who had eGFR <30 mL/min or eGFR <60 mL/min while remaining on tenofovir treatment. The Kidney Disease Outcomes Quality Initiative classification defines severe kidney impairment as eGFR <30 mL/min and moderate kidney impairment as 30–59 mL/min [36]. We used the MDRD equation for this analysis as there is some evidence that the MDRD equation is more sensitive at detecting eGFR <60 mL in our population [23,24]; and the MDRD equation is used to report eGFR results in our setting, so is the basis for clinical decisions such as drug dose adjustment or stopping tenofovir. We used a Cox regression model to assess associations between eGFR <30 mL/min and eGFR <60 mL/min on tenofovir treatment and baseline eGFR, age, baseline CD4 count, baseline weight, and concomitant protease inhibitor use. Because the effects of some of the predictors on the outcomes were not constant over time, we split the follow-up period into 0–6 months and >6 months after treatment initiation. We censored patients at their first occurrence of eGFR <30 or <60 mL/min; time of stopping tenofovir; death; or their last visit before database closure. We used Stata 13.0 for the statistical analyses [37].

The University of Cape Town Faculty of Health Sciences Human Research Ethics Committee approved the study (reference number 576/2011). The research ethics committees of the University of Cape Town and the University of the Witwatersrand approved the sites’ contribution of data to the study.

## Results

We included 15 156 patients. Their characteristics at ART initiation are summarized in Tables 1 and 2. The most common concomitant antiretrovirals were lamivudine (14 199 patients, 93.7%) and efavirenz (13 554 patients, 89.4%). Most patients (14 753 (97.3%)) started tenofovir in 2010 or later; 1 591 (10.5%) started in 2013 (when the CD4 count threshold for ART eligibility was increased). Median duration of follow-up on tenofovir was 12.9 months (interquartile range (IQR) 5.1–23.3).

**Table 1.** Characteristics at ART initiation of adult patients starting tenofovir-containing ART from two South African clinics

Characteristic	All patients	Baseline and subsequent eGFR available	
	n = 15 156	MDRD & CKD-EPI n = 11 112	Cockcroft-Gaultn = 9 283
Males (n, %)	5 688 (37.5)	4 065 (36.6)	3 391 (36.5)
Age (years) (median, IQR)	35.4 (29.9–42.0)	35.5 (30.0 to 42.0)	35.5 (30.1 to 41.9)
CD4 count (cells/µL) (median, IQR)	168 (83–243)^1^	173 (90 to 244)^2^	170 (88 to 241)^3^
Weight (kg) (median, IQR)	63.3 (55.6–73.5)^4^	64.1 (56.3 to 74.4)^5^	64.1 (56.3 to 74.3)

CKD-EPI: Chronic Kidney Disease Epidemiology Collaboration; MDRD: Modification of Diet in Renal Disease; IQR: interquartile range.

Data were not available in all patients: 1. n = 12 064; 2. n = 9 129; 3. n = 7 707; 4. n = 14 290; 5. n = 10 752.

**Table 2.** Kidney function at ART initiation of adult patients starting tenofovir-containing ART with baseline eGFR available from two South African clinics

Characteristic		MDRD	CKD-EPI	Cockroft-Gault
All patients				
n		13 396	13 396	11 868
eGFR (mL/min) (median, IQR)		98.6 (84.4–115.6)	110.4 (96.9–119.6)	90.2 (74.5–108.9)
Baseline eGFR category (n, %)	>90	8 769 (66)	11 138 (83)	5 960 (50)
	60–89	4 221 (32)	2 065 (15)	4 959 (42)
	30–59	383 (3)	177 (1)	928 (8)
	15–29	18 (0.1)	12 (0.1)	18 (0.2)
	<15	5 (0.04)	4 (0.03)	3 (0.03)
Baseline and subsequent eGFR				
n		11 112	11 112	9 283
eGFR (mL/min) (median, IQR)		98.5 (84.5–115.1)	110.4 (97.1–119.6)	91.1 (75.6–109.9)
Baseline eGFR category (n, %)	>90	7 273 (66)	9 283 (84)	4 816 (52)
	60–89	3 541 (32)	1 701 (15)	3 803 (41)
	30–59	286 (3)	120 (1)	654 (7)
	15–29	12 (0.1)	8 (0.1)	10 (0.1)
	<15	0	0	0

CKD-EPI: Chronic Kidney Disease Epidemiology Collaboration; MDRD: Modification of Diet in Renal Disease.

In those patients with baseline plus at least one subsequent eGFR available, predicted mean eGFR decreased slightly over time when eGFR was calculated using the MDRD or CKD-EPI equations. However, predicted mean eGFR increased over time when eGFR was calculated using the Cockcroft-Gault equation (Table 3).

**Table 3.** Predicted eGFR change from baseline in patients on tenofovir-containing ART from two South African clinics

Change from baseline (mL/min) Mean (95% CI)	3 months	6 months	12 months	24 months
MDRD	−0.3 (−0.4 to −0.2)	−0.7 (−0.8 to −0.5)	−1.3 (−1.7 to −1.0)	−2.6 (−3.3 to −1.9)
n^1^	9 295	5 234	5 537	2 486
CKD-EPI	−0.3 (−0.3 to −0.2)	−0.5 (−0.6 to −0.4)	−1.0 (−1.2 to −0.8)	−2.0 (−2.5 to −1.6)
n^1^	9 295	5 234	5 537	2 486
Cockcroft-Gault	0.8 (0.7 to 0.9)	1.6 (1.4 to 1.8)	3.3 (2.9 to 3.6)	6.5 (5.8 to 7.3)
n^1^	7 405	3 165	4 446	1 632

CI: confidence interval; CKD-EPI: Chronic Kidney Disease Epidemiology Collaboration; MDRD: Modification of Diet in Renal Disease.

1. Patients who were on tenofovir at the relevant time point, and had at least one eGFR value available within 0.5–4.0 months (3 month time point); 4.1–8.0 months (6 month time point); 8.1–18.0 months (12 month time point); and 18.1–30.0 months (24 month time point). The linear mixed effects model used all eGFR data from all relevant patients to predict changes from baseline.

**Figure 1.**
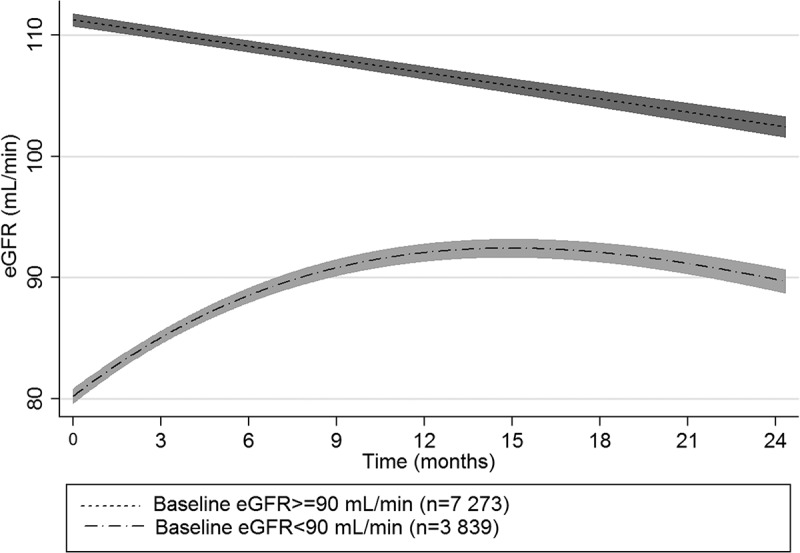
Predicted mean (95% confidence intervals) eGFR (Modification of Diet in Renal Disease) over time in patients on tenofovir-containing ART from two South African clinics.

In the subgroup of patients with eGFR <90 mL/min at baseline, eGFR improved over time (Table 4 and Figure 1). eGFR changes over time were similar in a sensitivity analysis restricted to patients who remained on tenofovir for at least 1 year (Table S1). In the 298 patients with baseline eGFR (MDRD) <60 mL/min, and in the 12 patients with baseline eGFR <30 mL/min, mean eGFR change from baseline at 12 months was 24.7 (95% CI 21.2–28.2) and 79.9 mL/min (95% CI 62.6–97.2), respectively.

**Table 4.** Predicted eGFR change from baseline according to baseline kidney function in patients on tenofovir-containing ART from two South African clinics

Change from baseline Mean (95% confidence interval)	3 months	6 months	12 months	24 months
MDRD				
Baseline eGFR ≥90 mL/min	−1.1 (−1.2 to −1.0)	−2.2 (−2.4 to −2.0)	−4.4 (−4.9 to −4.0)	−8.9 (−9.8 to −7.9)
n^1^	6 033	3 418	3 607	1 558
Baseline eGFR <90 mL/min	4.9 (4.5 to 5.3)	8.4 (7.8 to 9.1)	11.9 (11.0 to 12.7)	9.5 (8.4 to 10.5)
n^1^	3 262	1 816	1 930	928
CKD-EPI				
Baseline eGFR ≥90 mL/min	−0.6 (−0.6 to −0.5)	−1.1 (−1.3 to −1.0)	−2.3 (−2.5 to −2.1)	−4.6 (−5.1 to −4.2)
n^1^	7 731	4 413	4 621	2 034
Baseline eGFR <90 mL/min	6.2 (3.8 to 6.7)	10.6 (9.8 to 11.4)	14.6 (13.5 to 15.7)	10.7 (9.4 to 12.0)
n^1^	1 564	821	916	452
Cockcroft-Gault				
Baseline eGFR ≥90 mL/min	0.2 (0.01 to 0.3)	0.3 (0.02 to 0.6)	0.6 (0.04 to 1.2)	1.3 (0.1 to 2.5)
n^1^	3 800	1 669	2 352	817
Baseline eGFR <90 mL/min	4.0 (3.7 to 4.3)	7.1 (6.6 to 7.6)	11.0 (10.3 to 11.7)	12.6 (11.7 to 13.5)
n^1^	3 605	1 496	2 094	815

CI: confidence interval; CKD-EPI: Chronic Kidney Disease Epidemiology Collaboration; MDRD: Modification of Diet in Renal Disease.

1. Patients who were on tenofovir at the relevant time point, and had at least one eGFR value available within 0.5–4.0 months (3 month time point); 4.1–8.0 months (6 month time point); 8.1–18.0 months (12 month time point); and 18.1–30.0 months (24 month time point). The linear mixed effects model used all eGFR data from all relevant patients to predict changes from baseline.

Overall, 292/15 156 patients (1.9%) had at least one eGFR <30 mL/min after baseline while on tenofovir treatment. The incidence of eGFR <30 mL/min on treatment was 15.3 (95% CI 13.7–17.2) per 1 000 person years. Median time to first eGFR <30 mL/min was 1.9 months (IQR 0.8–8.0). The incidence of eGFR <30 mL/min was slightly lower when restricted to patients who had a baseline plus at least one subsequent eGFR on tenofovir treatment available, and eGFR ≥30 mL/min at baseline (Table S2). Older age, baseline eGFR <60 mL/min, lower CD4 count, and lower weight (Table 5) were associated with increased risk of developing eGFR <30 mL/min in the first 6 months of therapy. After 6 months, concomitant protease inhibitor therapy (compared to non-nucleoside reverse transcriptase inhibitor) and older age were associated with increased risk of developing eGFR <30 mL/min. After 6 months, the association with baseline CD4 count was attenuated and there was no significant association with baseline eGFR or weight.

**Table 5.** Associations with development of eGFR <30 mL/min in patients on tenofovir-containing ART from two South African clinics during the first 6 months on ART and after 6 months on ART

			0–6 months		>6 months	
Baseline characteristics		n^1^	Crude HR (95% CI)	Adjusted^2^ HR (95% CI) n = 10 283	Crude HR (95% CI)	Adjusted^2^ HR (95% CI) n = 10 283
Age	Per 10-year increase	14 633	1.5 (1.3 to 1.7)	1.5 (1.3 to 1.8)	1.4 (1.1 to 1.7)	1.6 (1.2 to 2.2)
eGFR (reference ≥60 mL/min)	<60 mL/min	13 003	7.2 (5.0 to 10.5)	4.6 (2.9 to 7.2)	1.5 (0.5 to 4.7)	1.3 (0.4 to 4.3)
CD4 (reference ≥200 cells/µL)	100–199 cells/µL	11 686	1.8 (1.1 to 2.9)	2.1 (1.2 to 3.5)	0.8 (0.4 to 1.5)	0.8 (0.4 to 1.6)
	50–99 cells/µL		3.7 (2.2 to 6.0)	4.1 (2.4 to 6.9)	1.3 (0.6 to 2.9)	1.2 (0.5 to 2.9)
	<50 cells/µL		6.2 (4.0 to 9.7)	7.9 (4.9 to 12.9)	2.4 (1.2 to 4.7)	3.1 (1.5 to 6.4)
Weight (reference ≥60 kg)	<60 kg	13 887	2.1 (1.6 to 2.8)	1.6 (1.1 to 2.1)	1.3 (0.8 to 2.0)	1.2 (0.7 to 2.2)
Concomitant ARV (reference NNRTI)	PI	14 571	1.9 (0.8 to 4.7)	2.0 (0.5 to 8.2)	2.8 (0.9 to 8.9)	6.1 (1.4 to 25.6)

1. Number of patients in the crude analyses. 2. Adjusted for treatment site and the other variables in the model. CI: confidence interval; eGFR: estimated glomerular filtration rate; HR: hazard ratio; NNRTI: non-nucleoside reverse transcriptase inhibitor; PI: protease inhibitor.

Overall, 1 085/15 156 patients (7.2%) had at least one eGFR <60 mL/min after baseline while on tenofovir treatment. The incidence of eGFR <60 mL/min on treatment was 58.9 (95% CI 55.5 to 62.5) per 1 000 person years. Median time to first eGFR <60 mL/min was 2.8 months (IQR 0.9 to 9.8). Older age, baseline eGFR <75 mL/min, lower CD4 count, lower weight and concomitant protease inhibitor use (compared to non-nucleoside reverse transcriptase inhibitor) were associated with increased risk of developing eGFR <60 mL/min in the first 6 months of therapy. After 6 months, there was no significant association with lower weight and concomitant protease inhibitor use (Table 6).

**Table 6.** Associations with development of eGFR<60 mL/min in patients on tenofovir-containing ART from two South African clinics during the first 6 months on ART and after 6 months on ART

			0–6 months		>6 months	
Baseline characteristics		n^1^	Crude HR (95% CI)	Adjusted^2^ HR (95% CI) n = 10 283	Crude HR (95% CI)	Adjusted^1^ HR (95% CI) n = 10 283
Age	Per 10-year increase	14 633	1.8 (1.7 to 1.9)	1.6 (1.5 to 1.7)	1.6 (1.5 to 1.8)	1.6 (1.4 to 1.9)
eGFR (reference ≥75 mL/min)	<75 mL/min	13 003	5.7 (4.9 to 6.7)	4.8 (4.0 to 5.8)	3.5 (2.7 to 4.4)	2.3 (1.7 to 3.1)
CD4 (reference ≥200 cells/µL)	100–199 cells/µL	11 686	1.4 (1.2 to 1.8)	1.5 (1.2 to 1.9)	1.1 (0.8 to 1.5)	1.1 (0.8 to 1.6)
	50–99 cells/µL		2.1 (1.7 to 2.7)	2.2 (1.7 to 2.8)	1.6 (1.1 to 2.3)	1.5 (1.0 to 2.3)
	<50 cells/µL		2.7 (2.1 to 3.4)	3.1 (2.4 to 4.0)	2.4 (1.7 to 3.4)	2.8 (1.9 to 4.0)
Weight (reference ≥60 kg)	<60 kg	13 887	1.3 (1.1 to 1.5)	1.3 (1.1 to 1.6)	1.1 (0.9 to 1.3)	1.0 (0.8 to 1.4)
Concomitant ARV (reference NNRTI)	PI	14 571	1.6 (1.0 to 2.7)	2.2 (1.1 to 4.2)	1.4 (0.6 to 3.1)	2.4 (0.8 to 7.6)

1. Number of patients in the crude analyses. 2. Adjusted for treatment site and the other variables in the model. CI: confidence interval; eGFR: estimated glomerular filtration rate; HR: hazard ratio; NNRTI: non-nucleoside reverse transcriptase inhibitor; PI: protease inhibitor.

## Discussion

We found that patients on tenofovir from two South African clinics experienced small but significant declines in eGFR over time overall, when eGFR was estimated using the MDRD or CKD-EPI equations. However, eGFR increased after tenofovir initiation in patients commencing tenofovir containing-ART with eGFR <90 mL/min, regardless of which estimating equation was used. Older patients and those with lower weight and more advanced disease were at increased risk of decline in eGFR to below 30 mL/min, and use of protease inhibitors increased that risk, particularly after 6 months of tenofovir-containing ART.

Our finding that overall patients on tenofovir experienced declines in eGFR over time is similar to the changes in eGFR over time seen in several previous studies [4,5,10,12,18,38]. However, that pattern is not consistent across all previous studies and the clinical relevance of mild eGFR changes is uncertain. Although the meta-analysis by Cooper et al. found that eGFR over time was lower in patients on tenofovir than those on other antiretrovirals in 11 studies that calculated eGFR using the Cockcroft-Gault formula, there was no significant difference when they analyzed the six studies that used the MDRD formula [3]. Estrella et al. found that although HIV-infected women on tenofovir had lower eGFRs over time than uninfected controls, the rates of eGFR decline were similar [39]. A recent South African cohort study in patients with a low prevalence of renal dysfunction at baseline found that eGFR (Cockcroft-Gault) increased over time [7]. A study in 19 patients on tenofovir found that estimated (Cockcroft-Gault) but not measured GFR declined over time. The authors suggest that the decline in eGFR can be explained by inhibition of tubular creatinine excretion rather than glomerular dysfunction [40].

In the subgroup of patients who had moderate or severe kidney dysfunction at baseline, eGFR improved substantially on treatment, regardless of which estimating equation was used. Similar increases in eGFR over time in those with kidney dysfunction at baseline have been seen in previous studies, both in patients on ART in general (including tenofovir-containing regimens) [8,11,16], and those on tenofovir-containing regimens specifically [6,8]. Such improvements might be due to effective treatment of underlying HIV-associated nephropathy [19], but might also be due to resolution of other acute illnesses present at ART initiation.

In our study, older patients and those with lower baseline eGFR, lower CD4 count, lower body weight and concomitant protease inhibitor use were at increased risk of kidney toxicity, which concurs with previous studies [4,5,9,14–18]. Baseline eGFR <60 mL/min was one of the strongest predictors of at least one eGFR <30 mL/min on treatment, supporting the current South African guideline recommendation to avoid tenofovir in those with baseline eGFR <60 mL/min.

Our study has several limitations. Our data are from routine clinical care, and, as per local guidelines, creatinine concentrations were measured in patients on tenofovir only. We therefore have no control group of patients not on tenofovir with which to compare our results. Nevertheless, although many studies report an increased risk of kidney dysfunction with tenofovir compared to other antiretrovirals [4,13,38,41–45], several studies found that tenofovir was not associated with increased risk [16,46–48]. Few studies reported incidence of clinical renal failure on tenofovir; in those that did, patients often had additional risk factors such as concomitant nephrotoxic drugs or comorbidities [11–13]. We have no data on concomitant medication or comorbidities such as diabetes or hypertension, and limited data on concurrent illness. All of those factors might influence eGFR. We were unable to assess the association between baseline viral load and eGFR as local guidelines did not recommend measurement of viral load at baseline. We were also unable to assess the association between body mass index and eGFR as heights were recorded infrequently. The sites do not routinely collect data on acute hospital admissions, so we were unable to estimate the incidence of acute renal failure in our cohort. They also do not routinely monitor other measures of kidney function such as proteinuria or glycosuria.

Our observational cohort study shows that patients on tenofovir experienced small but significant decreases in eGFR over time when calculated using the MDRD and CKD-EPI equations, and small increases in eGFR over time when calculated using the Cockcroft-Gault equation. In the subgroup of patients with kidney dysfunction at baseline, eGFR increased on treatment, regardless of which estimating equation was used. Clinically significant decreases in eGFR were uncommon. The benefits of tenofovir treatment probably outweigh the risks, even in settings where monitoring kidney function for all patients on tenofovir is not feasible. With the likely increase in tenofovir use given WHO recommendations to treat all patients regardless of CD4 count, in resource-limited settings with limited access to laboratory testing, screening and monitoring guidelines should consider focusing on higher-risk patients.

## Notes

To access the supplementary material to this article please see Supplementary Files under Article Tools online.
