# Corrigendum

**DOI:** 10.7448/IAS.20.1.22066

**Published:** 2017-06-02

Regarding the paper ‘Changes in estimated glomerular filtration rate over time in South African HIV-1-infected patients receiving tenofovir: a retrospective cohort study’ by *Renee De Waal, Karen Cohen, Matthew P Fox, Kathryn Stinson, Gary Maartens, Andrew Boulle, Ehimario U Igumbor, Mary-Ann Davies.*

Published in *Journal of the International AIDS Society*, Citation: De Waal et al. *Journal of the International AIDS Soceity*, **20**:21317. http://www.jiasociety.org/index.php/jias/article/view/21317 |https://doi.org/10.7448/IAS.20.01/21317

There was an error in one of the eGFR estimating equations. This led to errors in tables 2, 3 and 4. The correct equation and revised tables are detailed below:

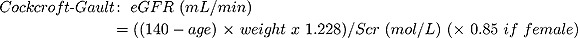

Therefore: Amongst those with a baseline and subsequent eGFR available, mean eGFR change from baseline at 12 months was –4.4 mL/min (95% Cl −4.9 to −4.0), −2.3 (−2.5 to −2.1), and 2.7 (2.2 to 3.2) in those baseline eGFR ≥90 mL/min; and 11.9 mL/min (11.0 to 12.7), 14.6 (13.5 to 15.7), and 17.8 (16.5 to 19.1), in those with baseline eGFR <90 mL/min, according to the MDRD, CKD-EPI (n = 11 112), and Cockcroft-Gault (n = 9 283) equations, respectively.

**Table 2.** Kidney function at ART initiation of adult patients starting tenofovir-containing ART with baseline eGFR available from two South African clinics

Characteristic		MDRD	CKD-EPI	Cockroft-Gault
All patients				
n		13 396	13 396	11 868
eGFR (mL/min) (median, IQR)		98.6 (84.4–115.6)	110.4 (96.9–119.6)	110.7 (91.4–133.7)
Baseline eGFR category (n, %)	>90	8 769 (66)	11 138 (83)	9 105 (77)
	60–89	4 221 (32)	2 065 (15)	2 527 (21)
	30–59	383 (3)	177 (1)	225 (2)
	15–29	18 (0.1)	12 (0.1)	9 (0.1)
	<15	5 (0.04)	4 (0.03)	2 (0.02)
Baseline and subsequent eGFR				
n		11 112	11 112	9 283
eGFR (mL/min) (median, IQR)		98.5 (84.5–115.1)	110.4 (97.1–119.6)	111.9 (92.8–134.9)
Baseline eGFR category (n, %)	>90	7 273 (66)	9 283 (84)	7 267 (78)
	60–89	3 541 (32)	1 701 (15)	1 865 (20)
	30–59	286 (3)	120 (1)	146 (2)
	15–29	12 (0.1)	8 (0.1)	5 (0.1)
	<15	0	0	0

CKD-EPI: Chronic Kidney Disease Epidemiology Collaboration; MDRD: Modification of Diet in Renal Disease.

**Table 3.** Predicted eGFR change from baseline in patients on tenofovir-containing ART from two South African clinics

Change from baseline (mL/min) Mean (95% CI)	3 months	6 months	12 months	24 months
MDRD	‒0.3 (‒0.4 to ‒0.2)	‒0.7 (‒0.8 to ‒0.5)	‒1.3 (‒1.7 to ‒1.0)	‒2.6 (‒3.3 to ‒1.9)
n^1^	9 295	5 234	5 537	2 486
CKD-EPI	‒0.3 (‒0.3 to ‒0.2)	‒0.5 (‒0.6 to ‒0.4)	‒1.0 (‒1.2 to ‒0.8)	‒2.0 (‒2.5 to ‒1.6)
n^1^	9 295	5 234	5 537	2 486
Cockcroft-Gault	1.0 (0.9 to 1.1)	2.0 (1.8 to 2.2)	4.0 (3.5 to 4.4)	8.0 (7.1 to 8.9)
n^1^	7 405	3 165	4 446	1 632

CI: confidence interval; CKD-EPI: Chronic Kidney Disease Epidemiology Collaboration; MDRD: Modification of Diet in Renal Disease. 1. Patients who were on tenofovir at the relevant time point, and had at least one eGFR value available within 0.5–4.0 months (3 month time point); 4.1–8.0 months (6 month time point); 8.1–18.0 months (12 month time point); and 18.1–30.0 months (24 month time point). The linear mixed effects model used all eGFR data from all relevant patients to predict changes from baseline.

**Table 4.** Predicted eGFR change from baseline according to baseline kidney function in patients on tenofovir-containing ART from two South African clinics

Change from baseline Mean (95% confidence interval)	3 months	6 months	12 months	24 months
MDRD				
Baseline eGFR ≥90 mL/min	‒1.1 (‒1.2 to ‒1.0)	‒2.2 (‒2.4 to ‒2.0)	‒4.4 (‒4.9 to ‒4.0)	‒8.9 (‒9.8 to ‒7.9)
n^1^	6 033	3 418	3 607	1 558
Baseline eGFR <90 mL/min	4.9 (4.5 to 5.3)	8.4 (7.8 to 9.1)	11.9 (11.0 to 12.7)	9.5 (8.4 to 10.5)
n^1^	3 262	1 816	1 930	928
CKD-EPI				
Baseline eGFR ≥90 mL/min	‒0.6 (‒0.6 to ‒0.5)	‒1.1 (‒1.3 to ‒1.0)	‒2.3 (‒2.5 to ‒2.1)	‒4.6 (‒5.1 to ‒4.2)
n^1^	7 731	4 413	4 621	2 034
Baseline eGFR <90 mL/min	6.2 (3.8 to 6.7)	10.6 (9.8 to 11.4)	14.6 (13.5 to 15.7)	10.7 (9.4 to 12.0)
n^1^	1 564	821	916	452
Cockcroft-Gault				
Baseline eGFR ≥90 mL/min	0.7 (0.5 to 0.8)	1.4 (1.1 to 1.6)	2.7 (2.2 to 3.2)	5.4 (4.3 to 6.5)
n^1^	3 800	1 669	2 352	817
Baseline eGFR <90 mL/min	6.6 (6.0 to 7.2)	11.7 (10.7 to 12.6)	17.8 (16.5 to 19.1)	18.8 (17.2 to 20.4)
n^1^	3 605	1 496	2 094	815

CI: confidence interval; CKD-EPI: Chronic Kidney Disease Epidemiology Collaboration; MDRD: Modification of Diet in Renal Disease. 1. Patients who were on tenofovir at the relevant time point, and had at least one eGFR value available within 0.5–4.0 months (3 month time point); 4.1–8.0 months (6 month time point); 8.1–18.0 months (12 month time point); and 18.1–30.0 months (24 month time point). The linear mixed effects model used all eGFR data from all relevant patients to predict changes from baseline.

